# Embedding equity and diversity principles in a complex multinational setting: methods, tools, capacity development and experiences from the first year of the Joint Action on Cardiovascular Diseases and Diabetes (JACARDI)

**DOI:** 10.1136/bmjgh-2025-019829

**Published:** 2025-11-16

**Authors:** Natalia Skogberg, Teresa Spadea, Benedetta Armocida, Jelka Zaletel, Beatrice Formenti, Ane Fullaondo, Yhasmine Hamu, Maria Nousiainen, Sinikka Kytö, Laura Musta, Chiara Di Girolamo, Janne Sorensen, Idil Hussein, Graziano Onder, Richard H Osborne

**Affiliations:** 1 Finnish Institute for Health and Welfare, Helsinki, Uusimaa, Finland; 2 ASL TO3 Epidemiologia, Collegno (TO), Piedmont, Italy; 3 Istituto Superiore di Sanità, Roma, Italy; 4 National Institute of Public Health, Ljubljana, Slovenia; 5 Fondazione Policlinico Universitario Agostino Gemelli IRCCS, Rome, Lazio, Italy; 6 Biosistemak Institute for Health Systems Research, Basque country, Spain; 7 Network for Research on Chronicity, Primary Care, and Health Promotion (RICAPPS), Basque country, Spain; 8 Department of Clinical and Biological Sciences, University of Turin, Turin, Piedmont, Italy; 9 University of Copenhagen, Copenhagen, Capital Region of Denmark, Denmark; 10 Fondazione Policlinico Gemelli IRCCS, Rome, Italy; 11 Universita Cattolica del Sacro Cuore, Rome, Italy; 12 La Trobe Rural Health School, La Trobe University, La Trobe, Victoria, Australia

**Keywords:** Health systems, Health policy, Accountability, Diabetes, Cardiovascular disease

## Abstract

Reducing social inequities in health requires acknowledgement of the deeply embedded intersectional nature of systemic drivers of these inequities. With increasing population cultural and ethnic diversity, it is essential to embed principles of equity, diversity and inclusion from the outset of programmes and policy development, thereby reducing the need for costly corrective measures. This practice paper outlines the design and first-year experiences of embedding equity and diversity principles in the European Union co-funded Joint Action on Cardiovascular Diseases and Diabetes (JACARDI). JACARDI involves 21 countries, 76 partners and 142 pilot projects, systematically integrating equity, diversity and inclusion across all activities. To support a shared understanding among partners on why equity and diversity are important and what are the key principles to consider during implementation, a conceptual 4Cs framework (critical reflection, context and data, co-design, communication) was developed. The framework was operationalised through a shared terminology glossary, integration of an equity and diversity lens within the JACARDI harmonised XV-step implementation methodology and an equity and diversity maturity matrix to guide and assess implementation. Uptake of these was systematically supported with capacity development. The developed methods and tools are designed to be transferable and scalable, with potential for supporting sustainable and inclusive health policy and practice across diverse contexts.

**WHAT IS ALREADY KNOWN ON THE TOPIC**

- With increasing population diversity, there is an acute need for development and implementation of diversity inclusive policy and practice to effectively and sustainably tackle social inequities in health.

**WHAT THIS STUDY ADDS**

- This practice paper outlines the development of novel methods and practical tools for systematically embedding equity, population diversity and inclusivity principles in a multinational European project involving 21 countries and 142 pilot projects that aim to reduce the burden of cardiovascular diseases and diabetes.

**HOW THIS STUDY MIGHT AFFECT RESEARCH, PRACTICE AND POLICY**

- The methods and tools presented in this paper can be used as such or adapted to other projects and contexts. To our awareness, this is the first project of its scale to develop and test such methods in a structured and systematic manner.

## Introduction

 Health inequities persist in Europe, with particularly pertinent social inequities in cardiovascular diseases and diabetes (CVD).^1^ Macrolevel structural and societal factors are the underlying mechanisms for health inequities, reinforcing unequal distribution of power, status and resources in the society^2 3^ surpassing the effect of microlevel factors, such as health behaviours, health service access and disease management.^4 5^

Decades of public health efforts have focused on reducing gender, educational, occupational and income-related health inequities.^6^ Simultaneously, key social determinants of health like race, ethnicity and migrant status/background, and structural racism and discrimination, have been rendered secondary or omitted altogether. This is despite evidence on their significant role as independent determinants and their complex interaction with other social determinants.^7 8^

The COVID-19 pandemic brought into the forefront the deep-rooted structural inequities and their intersectional role in generating and perpetuating social disadvantage.^9 10^ It also highlighted the consequences of siloed and fragmented health equity work.^11^ Key lessons learnt were the critical role of diversity inclusive health equity policy and practice for societal security and cohesion and the acute need for embedding population diversity principles in all health equity measures.^10 12^ Importance of appropriate data, defined strategy and resources,^13^ accessible and inclusive communications^14 15^ and co-design^14 16^ were also recognised.

In response to the evident need for diversity-inclusive equity-driven practice, the European Union (EU) cofunded Joint Action on Cardiovascular Diseases and Diabetes (JACARDI, November 2023–October 2027) places equity, diversity and inclusivity (referred to as equity and diversity from hereon) as a cross-cutting theme to achieve JACARDI’s general objective of reducing the burden of CVD and diabetes in Europe.^17^ With focus on implementing evidence-based actions, 66-million-euro budget, involvement of 76 partner organisations across 21 European countries and 142 pilot projects (later pilots), JACARDI has strong policy relevance and opportunity for sustainable impact at individual and structural levels. JACARDI serves as a unique platform for developing, applying and evaluating methods and tools for embedding equity and diversity principles in a complex practical setting.

This practice paper describes the initial process of embedding equity and diversity perspectives as well as developed methods, practical tools and capacity development during the first year of JACARDI. Embedding is contextualised through the four domains of Normalisation Process Theory (NPT)^18^: coherence (sense-making), cognitive participation (engagement), collective action (implementation) and reflexive monitoring (formal and informal evaluation). While equity-focused implementation science frameworks (e.g., the Health Equity Implementation Framework,^19^ equity-adapted ^20^Consolidated Framework for Implementation Research (CFIR) and Reach, Effectiveness, Adoption, Implementation and Maintenance (RE-AIM) RE-AIM extensions)^21^ also offer valuable perspectives on equity impacts and implementation determinants, NPT was selected for describing initiation of the embedding processes for its focus on the social and cognitive mechanisms through which new methods and tools are adopted, enacted and sustained by implementers.

## Setting the foundation

JACARDI targets six thematic areas: health literacy and awareness; data and registries; population screening; integrated care pathways; self-management and labour participation. The 142 pilots operate under one of these thematic areas (work packages) and implement evidence-based interventions in diverse public health and health service settings. All JACARDI pilots follow a harmonised Methodological framework comprising XV steps. The XV-step methodology is grounded on implementation science frameworks and follows the quality improvement model Plan-Do-Study-Act cycle.^22^

Following extensive discussions and advocacy work (coherence, cognitive participation), equity and diversity principles were deliberately embedded within the XV-step methodology through:

- Development of the 4Cs framework (critical reflection, context and data, co-design and inclusive and accessible communication)
- An equity and diversity lens
- Terminology glossary
- Equity and diversity maturity matrix
- Capacity development

Additionally, equity and diversity principles were incorporated in JACARDI communication and dissemination through practical tools and capacity development on inclusive and accessible communication.

The majority (61%) of the 142 pilots target a specific population group (e.g., persons with diabetes/CVD, groups in vulnerable situations, pregnant women and children, professionals). Early in JACARDI, thematic work packages included an open-ended question on how equity and diversity will be addressed in their pilot mapping surveys, although there was heterogeneity across work packages in how this question was formulated. Pilots targeting a specific population group often mentioned service adaptation, with several elaborating on inclusive outreach strategies and accessible communication. Some mentioned intersectional factors, including age, gender, ethnicity and cultural background.

### Mapping barriers and facilitators and formulating the theory of change

Five focus group interviews with the entire leadership team (total n=24, interviewed group size 3–7 participants) were conducted at the start of JACARDI in November 2023 to map previous experiences and perceptions on barriers and facilitators for embedding equity and diversity principles. Semistructured interviews were conducted by two senior equity and diversity experts (female, cisgender, white, one of them of migrant background). Interviews lasted from 1 hour 45 min to 2 hour and were transcribed verbatim.

To inform early design of the dynamic embedding process, a rapid analysis following principles of inductive thematic analysis was conducted. One of the interviewers reviewed the transcripts to identify recurring patterns and themes. These were then discussed and consolidated with the second interviewer. All participants provided written informed consent. Ethics Committee of the Finnish Institute for Health and Welfare approved the conduct of the interviews.

The leadership group perceived consideration of cultural and ethnic diversity crucial for reducing health inequities, and most could identify topic relevance for their work in JACARDI. However, majority were doubtful whether cultural and ethnic diversity can be translated into practice, seeing these concepts as very broad and abstract. Participants expressed explicit need for shared understanding on what is meant by equity and diversity in JACARDI, practical tools and support in operationalising these for implementation.

**Figure 1:**
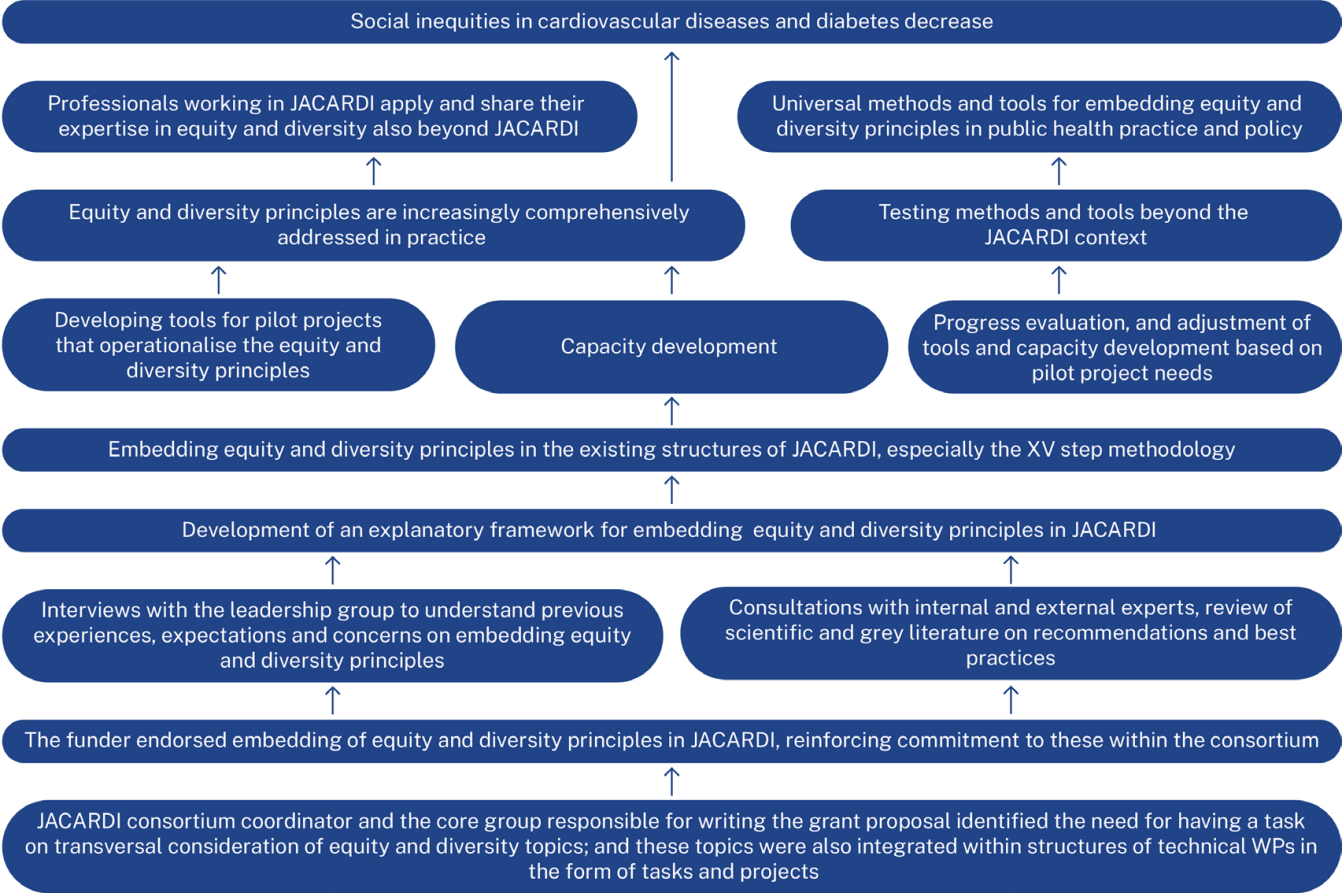
The theory of change for embedding equity and diversity principles in Joint Action on Cardiovascular Diseases and Diabetes (JACARDI).

Leadership interviews informed formulation of the theory of change for embedding equity and diversity principles in JACARDI (figure 1). The theory of change identifies prerequisites (endorsement from those who have the power to influence implementation and ensuring cognitive engagement of implementers), key enablers (methods, operationable tools, capacity development and adaptability of these to better meet the needs of implementers) and pathways through which developed methods, tools and capacity development can serve to reach the ultimate goal to reduce social inequities in CVD and diabetes in Europe (testing and further developing methods and tools and benefiting from the expertise of implementers beyond JACARDI).

## Putting theory into action

To establish a shared understanding (coherence) across all partners on why embedding equity and diversity is important and what are the key principles to consider, a conceptual framework was developed. The framework was operationalised with tools that supported pilot implementation (collective action). Additionally, comprehensive capacity development activities were introduced to initiate engagement (cognitive participation) of all partners.

### Conceptual framework

The conceptual framework on embedding equity and diversity draws from theory, review of scientific literature and grey literature describing good practices and recommendations, consultations with external experts and extensive expertise within JACARDI. The theoretical base originates from the WHO Commission on social determinants of health^23^ framework, the Diderichsen model for social basis of disparities in health,^24 25^ the theory of intersectionality,^3 26^ the life course perspective^27^ and the commercial determinants of health model.^28^ It also builds on existing equity-informed implementation research frameworks,^29 30^ which provide important foundations for embedding equity in implementation science.

**Figure 2:**
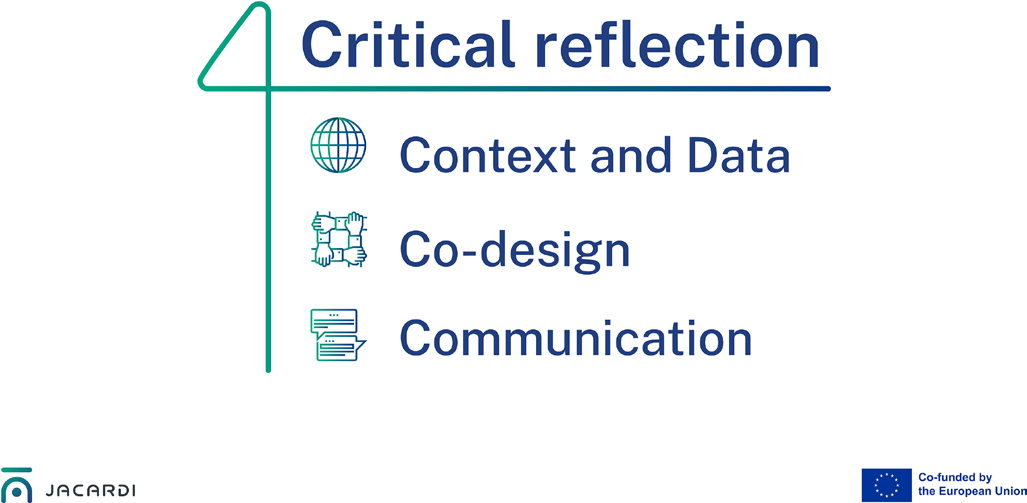
The 4C conceptual framework on equity and diversity in JACARDI (online supplemental file 1). Equity and diversity maturity matrix (online supplemental file 2). Pilot responses to the equity and diversity maturity matrix steps I–VI. 4C, critical reflection, context and data, co-design, communication; JACARDI, Joint Action on Cardiovascular Diseases and Diabetes.

The framework emphasises the contextual role of macrolevel factors in generation and perpetuation of social inequities in health, the intersectional nature of social identities and vulnerabilities, as well as the complex interaction between macrolevel and individual characteristics, such as age, sex, gender, ethnicity and migrant origin. In acknowledgement of the need for simplicity and operability raised during interviews with the leadership group, the framework was summarised with an easily recallable 4Cs acronym: critical reflection, context and data, co-design and communication (figure 2).

The 4Cs framework is designed to complement, not replace, existing equity-oriented implementation science frameworks. It provides a practice-oriented tool to operationalise equity and diversity principles in JACARDI’s large-scale, multicountry context, serving as an accessible entry point for practitioners with varying levels of expertise in equity and implementation science.

The foundation of the 4C framework lies within application of *critical reflection*. Also referred to as cultural humility or critical consciousness, critical reflection involves reflective sense-making (coherence) through continuous examination of own biases, stereotypes, privilege and power.^31 32^ In essence, application of critical reflection facilitates recognition of the deeply embedded invisible societal structures that shape and reinforce social inequities in health.^33 34^ Developing capacity (cognitive participation) of health professionals in application of critical reflection contributes towards social justice through influencing practice (collective action), supporting dignity, autonomy and equitable delivery of high-quality care to all, regardless of sex, gender, race, ethnicity, socioeconomic status and other social characteristics.^32^

The 4C framework identifies three key phases of implementation (collective action), where critical reflection should be applied, namely when identifying priority areas of focus, planning and roll-out of actions as well as dissemination through informing the public and other relevant stakeholders on policy and practice-related plans, services, developments and activities.

The framework advocates for application of critical reflection when making decisions on priority areas of focus as well as during planning and roll-out of actions through a comprehensive analysis of *context and data*. Social and economic context shapes power, privilege and resource distribution and influences an individual’s identity, social position and health.^3^ Analysis of context should involve a comprehensive consideration of the macrolevel social, economic, environmental and political factors, which shape and reinforce unequal distribution of power, status and resources.^23 35^ Critical reflection should also be applied during impact assessment, considering who is impacted, how and whether there are participation barriers for some groups.^36^

Quantitative and qualitative data are essential for informing policy and practice.^11 37^ Professionals responsible for data collection and health monitoring hold a great deal of power, since their decisions on how and what data are collected and presented play an essential role in shaping the foundations for evidence-based policy and practice. Data availability also determines which groups, phenomena and health indicators are highlighted, and alternatively which groups or phenomena may be rendered invisible through data limitations.^38^ Critical reflection should be applied to identify data gaps and biases,^36^ following which supplementary data can be collected.

Key stakeholders should be appropriately engaged in all phases of implementation through *co-design*. Co-design utilises a bottom-up approach through involvement of end-users and end-beneficiaries, who are often under-represented and insufficiently heard in policy and practice.^39 40^ Critical reflection should be applied to avoid tokenism and epistemic injustice, which are typical pitfalls of research with groups at greater risk of vulnerabilities.^41^ End-users and other key stakeholders should be involved at all stages, from formulation of the problem to dissemination of the results, including returning knowledge back to the community.^42^ Co-design is essential for building trust and ensuring acceptability, effectiveness and sustainability of the developed policies and interventions.^43^

*Communication* relates to sending and receiving verbal and non-verbal information and making meaning.^44^ Communication should be accessible to all population groups, and it should consider diversity of the audiences. In addition to being a basic human right,^45^ the imperative for inclusive and accessible communication is reinforced through, for example, the European Accessibility Act^46^ and Equality and Anti-Discrimination Laws.^47^ Inclusive and accessible communication requires critical reflection to ensure it suits everyone, regardless of abilities, gender, culture, ethnicity or life situation. This involves considering how the message is delivered, who decides what is communicated, the diverse meanings conveyed, who is reached and when and where the communication occurs.^48^ Use of interpreters and/or cultural mediators is essential to reduce social, cultural and linguistic barriers, which restrict communication between health professionals and clients, and thus affect the quality of care and patient outcomes.^49^

### Practical tools

A glossary was compiled to support a shared understanding (coherence) and harmonised use (collective action) of terminology. To initiate and support critical reflection among project partners from the very start of JACARDI, the concept of an equity and diversity lens was introduced. This lens provided examples and supporting questions for considering equity and diversity principles within the JACARDI XV-step Methodological framework, further strengthening coherence and cognitive participation of implementers.

An equity and diversity maturity matrix was developed as a practical tool to support pilot implementation (collective action). Thematic work package leaders and pilot implementers were engaged (cognitive participation) in the development of the maturity matrix through asking for feedback on matrix drafts. The matrix was also presented at scientific seminars; received feedback related to clarifying the rationale, how and when it will be used as well as wording and terminology.

The matrix operationalises the 4C principles through further examples on how equity and diversity lens can be applied at different levels of comprehensiveness within the JACARDI XV-step methodology (see table 1 for the outline and online supplemental 1 for the full version of the maturity matrix). The maturity matrix was designed to be easily understood by its users, including those with limited experience in equity and diversity (coherence).

**Table 1:** JACARDI XV-step methodology and the equity and diversity maturity matrix outline

Steps	Maturity matrix substeps translating equity and diversity principles into practice
I: JACARDI core pilot team	1.1 Compose a diverse core pilot team
	1.2. Strengthen capacity in equity and diversity within the core pilot team
II: Definition of the problem and the general objective	2.1 Apply equity and diversity principles in the definition of the problem and the general objective
III: Situation analysis at the site of the implementation including key stakeholder analysis	3.1 Conduct a pilot-level stakeholder analysis
	3.2 Meaningfully engage diverse stakeholders
	3.3 Identify the impact on diverse end users/ end beneficiaries
IV: Refinement of the general objective	4.1 Engage diverse stakeholders in refinement of the general objective
V: Definition of specific objectives	5.1 Apply the equity and diversity perspectives in the definition of specific objectives
	5.2 Consider equity and diversity when selecting EU best practices/other evidence-based practices
VI: Pilot implementation plan number 1	6.1 Engage diverse stakeholders in development of the pilot implementation plan
	6.2 Integrate equity and diversity perspectives in the pilot implementation plan
	6.3 Integrate equity and diversity perspectives in the pilot communication
VII: Roll-out of actions and monitoring	7.1 Identify how equity and diversity principles will be monitored
	7.2 Apply equity and diversity perspectives during roll-out of actions
	7.3 Continue strengthening capacity in equity and diversity within the pilot team
	7.4 Consider core pilot team composition
VIII: Intermediate report number 1	8.1 Engage diverse stakeholders in evaluation of intermediate results
	8.2 Integrate equity and diversity perspectives in intermediate reporting
	8.3 Integrate equity and diversity perspectives in intermediate reporting on pilot communication
IX: Pilot implementation plan number 2	9.1 Engage diverse stakeholders in development of pilot implementation plan
	9.2 Integrate equity and diversity perspectives in the pilot implementation plan
	9.3 Integrate equity and diversity perspectives in the pilot communication
X: Roll out of actions and monitoring	10.1 Monitor (and if needed revise) equity and diversity principles
	10.2 Apply equity and diversity perspectives during roll-out of actions
	10.3 Continue strengthening capacity in equity and diversity within the pilot team
	10.4 Consider core pilot team composition
XI Intermediate report number 2	11.1 Engage diverse stakeholders in evaluation of intermediate results
	11.2 Integrate equity and diversity perspectives in intermediate reporting
	11.3 Integrate equity and diversity perspectives in intermediate reporting on pilot communication
XII Final implementation report	12.1 Return results to the community and engage diverse stakeholders in evaluation of results
	12.2 Integrate equity and diversity perspectives in reporting
	12.3 Integrate equity and diversity perspectives in reporting on pilot communication
XIII Focus on the key stakeholders’ engagement in building sustainability	13.1 Revisit (and if needed revise) the key stakeholder analysis
	13.2 Apply equity and diversity perspectives in dissemination of results and sustainability actions
	13.3 Apply inclusivity and accessibility guidelines in material presented to the stakeholder board
XIV Sustainability action plan	14.1 Apply equity and diversity perspectives in development of the sustainability action plan
	14.2 Integrate equity and diversity perspectives in the sustainability action plan
	14.3 Integrate inclusive and accessible communications in the sustainability action plan
XV Celebrate the success	15.1 Engage diverse stakeholders in planning final dissemination events
	15.2 Consider representation among the speakers in the final dissemination events
	15.3 Apply the principles of inclusive and accessible communications in final dissemination events

EU, European Union; JACARDI, Joint Action on Cardiovascular Diseases and Diabetes.

Pilots are advised to use the maturity matrix both as a guiding tool for pilot planning and self-evaluation, thus supporting implementation at a practical level (collective action) and capacity development (cognitive participation). The matrix outlines three levels of maturity: approaching, meeting and exceeding. The approaching level involves active application of critical reflection. Whether pilots reach meeting or exceeding levels depend on previous experiences and level of engagement of implementors as well as available resources.

Pilots implemented the first six steps of the JACARDI methodology during the first year, with 124 (87%) pilots completing the maturity matrix for these steps by February 2025 (collective action). The highest proportion of meeting or exceeding answers was reported for application of equity and diversity principles in general objective definition, stakeholder analysis and engagement of diverse stakeholders in general objective refinement and implementation plan development (online supplemental 2). Approaching was most frequently reported for meaningful engagement of diverse stakeholders, considering equity and diversity principles when selecting evidence-based projects to guide implementation and pilot communication.

Information on the level of achieved maturity will be gathered annually. Structured questionnaires to all pilots, supplemented with qualitative interviews with a smaller number of implementers, will be gathered midway and at the end of JACARDI to assess the embedding process and the feasibility, acceptability, usability and comprehensiveness of the maturity matrix (reflexive monitoring).

### Capacity development

A range of capacity development activities were introduced during the first year to further strengthen a shared understanding across partners (coherence), engagement (cognitive participation) and pilot implementation (collective action). Capacity development activities included practical application, didactic activities, mentorship and expert consultations, knowledge sharing and technical assistance^50^ (table 2). Masterclasses on the 4C principles formed a foundation for a shared understanding on equity and diversity across partners, and these are described in further detail in table 3, including the feedback gathered from the participants (reflexive monitoring).

**Table 2:** Capacity development on equity and diversity during the first year of JACARDI

Type of activity	Format	Description
Didactic activities	60 s on equity and diversity	Bite-sized concepts related to equity and diversity, often through storytelling and/or use of visuals. Altogether, 14 presentations were held at the start of regular leadership meetings.
	Master classes	Four masterclasses, each 1.5 hours in length, held between January and February 2023. Held via Teams and recorded for later use, introducing the 4Cs framework on equity and diversity.
	Presentations	Regular presentations at consortium webinars and in-person meetings, and leadership meetings, and communication meetings.
Mentorship and expert consultations	One-on-one consultations	One to two meetings held with each of the six thematic area leaders held during spring 2024 on how the equity and diversity principles can be applied in practice to their specific thematic area.
	Tailored thematic consultations	Regular ad hoc consultations on inclusivity and accessibility of communications, and bimonthly communication review.
Practical application	Thematic workshops	Three workshops on inclusive and accessible communications with thematic communication experts.
	Use of developed tools	Active use of the maturity matrix and terminology glossary contribute to capacity development across pilots. Communication experts are advised to use the guidelines for inclusive and accessible communications.
Knowledge sharing	Focus group interviews and regular meetings with the leadership group	Equity and diversity principles as central theme in the focus group interviews with JACARDI leadership group and at several methodological meetings. Regularly raised also during other meetings.
Technical assistance	Equity and diversity maturity matrix introductory workshops	Six workshops with pilots, introducing the maturity matrix. Participants started filling out the matrix during the workshop and could ask any questions they had. Possibility to ask also via e-mail.

4C, critical reflection, context and data, co-design; JACARDI, Joint Action on Cardiovascular Diseases and Diabetes.

**Table 3:** Masterclasses introducing the key principles of equity and diversity in JACARDI

Topic and trainer	Learning objectives	Participated (gave feedback)	Feedback score (1–5)	Other feedback
Critical reflection. Trainer: Dr. Janne Sørensen, JACARDI Scientific Advisory Board Member, University of Copenhagen (Denmark)	Awareness of one’s own sociocultural background, identity, implicit biases and tendency to stereotype, and how these impact doctor-patient interactions. Understanding how power structures reinforce social inequities and impact opportunities of different groups. Understanding intersections of patients’ characteristics and developing capacity in applying an intersectional approach to assess vulnerabilities, strengths and unique needs.	94 (57)	4.3	The thematic area was perceived as relevant, and both the masterclass arrangements and the speaker were perceived as good. However, it was expressed that the content should be more tailored to the JACARDI project, with added practical examples.
Co-design. Trainer: Professor Richard Osborne, health literacy thematic lead in JACARDI, La Trobe University (Australia).	Understanding that co-design requires authentic engagement with underserved communities and other stakeholders. Understanding that incorporation of diverse lived experiences strengthens interventions and programme development. Awareness that co-design and the grassroot vs top-down approach is essential for achieving meaningful and sustainable change.	97 (49)	4.4	The session was perceived as inspiring and clarified important concepts, with engaging participation and a captivating speaker. However, participants would have hoped for more practical examples and that the materials for the masterclass would have been available in advance.
Context and data. Trainers: Dr Natalia Skogberg, lead on equity and diversity; Finnish Institute for Health and Welfare (THL); Dr Teresa Spadea, co-lead on equity and diversity in JACARDI, Epidemiology Unit, ASL TO3	Understanding the role of social, political and environmental context in health inequities. Understanding the importance of health equity impact assessment to ensure that the developed measures are diversity inclusive. Understanding how measuring different indicators of socioeconomic position helps to explain the multiple and intersecting pathways to health inequalities and to identify policy targets and priorities	91 (30)	4.3	The content was perceived as clear, and the structure of the class was perceived to be effective in fostering an inspiring and active learning environment. Need for more depth on the topics, more practical examples and more time for discussions was expressed.
Inclusive and accessible communication. Trainer: Sinikka Kytö, co-lead of communication and dissemination, lead on inclusive and accessible communications in JACARDI; THL	Understanding that inclusive communication fosters equity, creativity and societal well-being by embracing diverse perspectives. Awareness that communications must be accessible, co-designed with the target audience, and use appropriate communication channels. Awareness of how language and images can reinforce stereotypes, what is inclusive language and importance of visuals that represent the target population.	84 (30)	4.4	The content was perceived as relevant and well-structured, and it was perceived to promote a fairer society through an effective and inclusive presentation style. Participants expressed a wish for more interactive activities, case studies, receiving materials in advance and less repetition.

JACARDI, Joint Action on Cardiovascular Diseases and Diabetes.

## Conclusion

During its first year, JACARDI focused on systematically embedding equity and diversity principles across a large-scale, multicountry initiative addressing CVD and diabetes. This involved developing methods, practical tools and capacity development to support shared understanding (coherence), partner engagement (cognitive participation) and implementation (collective action). The 4Cs conceptual framework, equity and diversity lens and maturity matrix operationalised broad equity and diversity principles, aiming to make them more accessible to partners with diverse expertise and contexts. Resourcing equity and diversity expertise and persistent advocacy were crucial for motivating partners and supporting uptake. Endorsement from leadership and continuous engagement of pilot implementors remains pivotal to ensure that embedding efforts move beyond superficial adoption.

Capacity development during the first year focused on introducing the 4C principles and supporting practical use of the equity and diversity maturity matrix. Pilot’s feedback on the need for more practical examples will be addressed, for example, through unstructured drop-in consultations and using JACARDI pilots as case studies during workshops.

The major strength of developed methods and tools is that these were designed together with the users for widely heterogeneous implementers, with diverse educational and professional backgrounds and level of previous engagement with equity and diversity topics, contexts and target groups. This diversity provides an invaluable opportunity to test and refine the methods, tools and processes across multiple contexts and population groups, from general populations to specific population groups at greater risk of health inequities.

Challenges included developing methods and tools while pilots were already initiating activities. Pilots had the opportunity to participate in the development of the maturity matrix, and regular feedback was gathered to support reflexive monitoring of the embedding process and to identify needs for modifications. However, direct input from end-users during formulation of the theory of change was absent. This would have likely further strengthened the theory of change and facilitated even greater implementer engagement. Systematic baseline data on implementers’ prior engagement with equity and diversity were not collected, as pilots expressed overwhelm by methodological and administrative complexity of JACARDI. However, some baseline insights were obtained from thematic work package pilot mapping surveys that included a question on equity and diversity.

In the upcoming years, ongoing engagement of pilot implementers, leadership and community stakeholders through gathering insights on their experiences of the embedding process will enhance the feasibility, usability and comprehensiveness of the developed methods and tools. Practical application of the methods and tools will also be monitored through analysing maturity matrix responses, case studies and measuring equity outcomes.

This paper focused on describing the methods, tools and capacity development for embedding equity and diversity principles that hold potential to enhance the comprehensiveness, effectiveness and sustainability of equity-oriented interventions within JACARDI. Developed methods and tools can also inform future public health initiatives across Europe, contributing to broader regional and global agendas on reducing inequities in non-communicable disease prevention and care. At practical level, these methods and tools can guide professionals in assessing the current level of equity integration and identifying concrete steps for strengthening inclusive approaches to prevention, diagnosis and management of CVD and diabetes. At policy level, these can help identify drivers of structural inequities, increase accountability and support benchmarking and monitoring of equity-related progress across diverse health systems, contributing to more liable and targeted resource allocation.

## Supplementary material

10.1136/bmjgh-2025-019829 online supplemental file 1

10.1136/bmjgh-2025-019829 online supplemental file 2

## Data Availability

Data are available upon reasonable request.
