# Supplementary material for: Embedding equity and diversity principles in a complex multinational setting: methods, tools, capacity development and experiences from the first year of the Joint Action on Cardiovascular Diseases and Diabetes (JACARDI)
Source: BMJ Glob Health. 2025 Nov 16;10(11):e019829. doi: 10.1136/bmjgh-2025-019829 (PMC12625843; doi:10.1136/bmjgh-2025-019829)
Supplement: online supplemental file 1 [file bmjgh-10-11-s001.pdf]

Supplement 1 Equity and diversity maturity matrix

Equity and diversity are cross-cutting themes in JACARDI. This equity and diversity maturity matrix is a practical tool for pilots, providing concrete examples for integration of equity and diversity perspectives at different levels of comprehensiveness. The steps in the matrix correspond with the XV steps of the JACARDI harmonised methodology for pilots. The equity and diversity matrix draws from the 4Cs principles identified as core components for integration of equity and diversity in JACARDI: Critical reflection; Context and data; Co-design; and inclusive and accessible Communications.

How to use the equity and diversity matrix?

The core pilot team will use the matrix to describe the pilot's consideration of equity and diversity. The different levels in the equity and diversity matrix provide concrete examples on how this can be done at different levels of comprehensiveness. Ideally, the pilots will use the matrix as a guiding tool when planning their activities. Pilots will report consideration of equity and diversity at the four key points of pilot implementation:

Steps I-VI: when compiling a pilot implementation plan (Webropol questionnaire, by M12)
Steps VII-VIII: intermediary reporting 1 (Integrated within general reporting)
Steps IX-XI: intermediary reporting 2 (Integrated within general reporting)
Step XII-XV: final reporting (Integrated within general reporting, by M48)

The steps in the equity and diversity matrix have three levels: approaching, meeting, exceeding. The pilots need to describe the level that best describes their activities. This will vary depending on the resources and previous experiences in equity and diversity perspectives among the core pilot team. For some, being at the approaching level will be an important step towards integration of equity and diversity, while some will reach the exceeding level. Use the comments field to provide additional information. If it is difficult to identify the appropriate level, a description of activities can be added in the open text field.

Pilot responses will be used to identify supporting activities for integrating equity and diversity perspectives, reporting to HADEA and at General Assemblies, and in publications related on equity and diversity in JACARDI.

Co-funded by
the European Union

Equity and diversity matrix outline:

STEP I: JACARDI core pilot team
1.1 Compose a diverse core pilot team
1.2. Strengthen capacity in equity and diversity within the core pilot team

STEP II: Definition of the problem and the general objective
2.1 Apply equity and diversity principles in the definition of the problem and the general objective

STEP III: Situation analysis at the site of the implementation including key stakeholder analysis
3.1 Conduct a pilot-level stakeholder analysis
3.2 Meaningfully engage diverse stakeholders
3.3 Identify the impact on diverse end users/ end beneficiaries

STEP IV Refinement of the general objective
4.1 Engage diverse stakeholders in refinement of the general objective

STEP V Definition of specific objectives
5.1 Apply the equity and diversity perspectives in the definition of specific objectives
5.2 Consider equity and diversity when selecting EU best practices/other evidence-based practices

STEP VI Pilot implementation plan No 1
6.1 Engage diverse stakeholders in development of the pilot implementation plan
6.2 Integrate equity and diversity perspectives in the pilot implementation plan
6.3 Integrate equity and diversity perspectives in the pilot communication

STEP VII Roll-out of actions and monitoring
7.1 Identify how equity and diversity principles will be monitored
7.2 Apply equity and diversity perspectives during roll-out of actions
7.3 Continue strengthening capacity in equity and diversity within the pilot team
7.4 Consider core pilot team composition

STEP VIII Intermediate report No 1
8.1 Engage diverse stakeholders in evaluation of intermediate results
8.2 Integrate equity and diversity perspectives in intermediate reporting
8.3 Integrate equity and diversity perspectives in intermediate reporting on pilot communication

IX Pilot implementation plan No 2
9.1 Engage diverse stakeholders in development of pilot implementation plan
9.2 Integrate equity and diversity perspectives in the pilot implementation plan
9.3 Integrate equity and diversity perspectives in the pilot communication

Co-funded by
the European Union

X Roll out of actions and monitoring
10.1 Monitor (and if needed revise) equity and diversity principles
10.2 Apply equity and diversity perspectives during roll-out of actions
10.3 Continue strengthening capacity in equity and diversity within the pilot team
10.4 Consider core pilot team composition

XI Intermediate report No 2
11.1 Engage diverse stakeholders in evaluation of intermediate results
11.2 Integrate equity and diversity perspectives in intermediate reporting
11.3 Integrate equity and diversity perspectives in intermediate reporting on pilot communication

XII Final implementation report
12.1 Return results to the community and engage diverse stakeholders in evaluation of results
12.2 Integrate equity and diversity perspectives in reporting
12.3 Integrate equity and diversity perspectives in reporting on pilot communication

XIII Focus on the key stakeholders' engagement in building sustainability
13.1 Revisit (and if needed revise) the key stakeholder analysis
13.2 Apply equity and diversity perspectives in dissemination of results and sustainability actions
13.3 Apply inclusivity and accessibility guidelines in material presented to the stakeholder board

XIV Sustainability action plan
14.1 Apply equity and diversity perspectives in development of the sustainability action plan
14.2 Integrate equity and diversity perspectives in the sustainability action plan
14.3 Integrate inclusive and accessible communications in the sustainability action plan

XV Celebrate the success
15.1 Engage diverse stakeholders in planning final dissemination events
15.2 Consider representation among the speakers in the final dissemination events
15.3 Apply the principles of inclusive and accessible communications in final dissemination events

Co-funded by the European Union

	Approaching	Meeting	Exceeding	Describe
**STEP I: JACARDI Core pilot team**				
**1.1. Compose a diverse core pilot team**	a) The core pilot team consists of professionals from **different disciplines and social groups**, representing at least two key characteristics of the community the pilot aims to serve.	b) The core pilot team consists of professionals from different disciplines and social groups (e.g. age, gender, ethnicity), and is **moderately representative** of the community the pilot aims to serve (i.e. 50% of key characteristics of the served community are represented within the team).	c) The core pilot team consists of professionals from diverse disciplines, social groups, and is **representative of the community** the pilot aims to serve (i.e. 75% or more of the key characteristics identified are represented within the team).	
**1.2. Strengthen capacity in equity and diversity within the core pilot team**	A) The core pilot team members **understand and can explain** the 4Cs principle and the relevance of equity and diversity in JACARDI to other partners and this is demonstrated through integrating equity and diversity perspectives in the pilot implementation plan.	b) The core pilot team members understand how to include equity and diversity perspectives in their pilot implementation but **need support** (e.g. consultations, workshops) of equity and diversity subject matter experts on how to apply them in practice.	c) The core pilot team members feel empowered to apply sufficiently concrete equity and diversity aspects in their implementation plans in an **autonomous** manner, and **actively seek opportunities to strengthen their capacity** in equity and diversity beyond JACARDI structures, for example through external seminars, trainings and independent readings.	
**Step II: Definition of the problem and the general objective**				
**2.1 Apply equity and diversity principles in the definition of the problem and the general objective**	a) Definition of the problem and general objective were defined based on **critical reflection** of available quantitative and qualitative data on population demographic, socioeconomic, and ethnic and cultural diversity.	b) In addition to using available quantitative and qualitative data, definition of the problem and the general objective involved **consultations** (e.g. workshops, interviews) with health professionals and end users.	c) In addition to using available quantitative and qualitative data, definition of the problem and the general objective involved meaningful engagement of health professionals and end users through **co-design**.	

Co-funded by the European Union

	Approaching	Meeting	Exceeding	Describe
**Step III: Situation analysis at the site of the implementation including key stakeholder analysis**				
**3.1 Conduct a pilot-level stakeholder analysis**	a) **Critical reflection** was applied to identify diverse stakeholders during the pilot-level stakeholder analysis conducted internally by the core pilot team.	b) **Consultations** were conducted with diverse individuals, groups, or entities considered to be potentially directly or indirectly affected by the pilot to comprehensively identify all relevant stakeholders.	c) The pilot level analysis was carried out in **co-design** with diverse individuals, groups, or entities considered directly or indirectly affected by the pilot.	
**3.2 Meaningfully engage diverse stakeholders**	a) The pilot-level **stakeholder board** was established together with stakeholders and is diverse in its composition (e.g. experts and professionals, policy makers representing different disciplines and social groups, and persons with lived experiences).	b) In addition to the pilot-level **stakeholder board**, groups of diverse end users/end beneficiaries, representative of the community the pilot wants to serve, was assembled for relevant consultations/workshops to identify the key themes and discourses that should be taken into account in the pilot.	c) In addition to the pilot-level **stakeholder board** and groups of diverse **end users/end beneficiaries,** the pilot flexibly assembled other **groups** on specific topics (e.g. constituting of specific professional groups or groups that have relevant insights in some current theme related to pilot implementation) to gain more comprehensive insights that benefit the pilot.	
**3.3 Identify the impact on diverse end users/ end beneficiaries**	a) Impact on diverse end users/ end users was identified through **critical reflection** on existing data and good practices.	b) Impact on diverse end users/ end beneficiaries was identified through critical review of existing data and good practices and **consultations** with diverse end users and other relevant stakeholders.	c) Impact on diverse end users/ end beneficiaries was identified through critical review of existing and new data and good practices, and meaningful engagement through **co-design** with diverse end users and other relevant stakeholders.	
**STEP IV Refinement of the general objective**				
**4.1 Engage diverse stakeholders in refinement of the general objective**	a) The general objective was refined with the pilot team, applying **critical reflection** in consideration of equity and diversity among the end users/ end beneficiaries.	b) The general objective was refined based on **consultations** with diverse stakeholders and end users/ end beneficiaries.	c) The general objective was refined in **co-design** with diverse stakeholders and end users/ end beneficiaries.	

Co-funded by the European Union

	Approaching	Meeting	Exceeding	Describe
**Step V Definition of specific objectives**				
**5.1 Apply the equity and diversity perspectives in the definition of specific objectives**	a) Specific objectives were defined within the pilot team, based on **critical reflection** on equity and diversity among the end users/ end beneficiaries.	b) Specific objectives were defined based on **consultations** with diverse end users/ end beneficiaries and other stakeholders.	c) Specific objectives were defined in **co-design** with diverse end users/ end beneficiaries and other stakeholders.	
**5.2 Consider equity and diversity when selecting EU best practices/other evidence-based practices**	a) **Critical reflection** considering equity and diversity perspectives was applied when selecting previous good practices (either from the EU Best Practices Portal or other evidence-based practices).	b) EU Best practices/other evidence-based practices were selected based on critical reflection and **consultations** with diverse stakeholders.	c) EU best practices/other evidence-based practices were selected based on critical reflection and **co-design** with diverse stakeholders.	
**STEP VI Pilot implementation plan No 1**				
**6.1 Engage diverse stakeholders in development of the pilot implementation plan**	a) Key stakeholders/ stakeholder board was **informed** on relevant parts of the pilot implementation plan.	b) Key stakeholders/stakeholder board was **consulted** when developing the pilot implementation plan.	c) Key stakeholders/stakeholder board was meaningfully engaged through **co-design** when developing the pilot implementation plan.	
**6.2 Integrate equity and diversity perspectives in the pilot implementation plan**	a) Equity and diversity perspectives were **integrated in the general objective** of the pilot implementation plan.	b) The pilot implementation plan had at least one **specific objective** related to equity and diversity.	c) The pilot implementation plan had **an action point** on equity and diversity under each specific objective.	

Co-funded by the European Union

	Approaching	Meeting	Exceeding	Describe
**6.3 Integrate equity and diversity perspectives in the pilot communication**	a) The pilot implementation plan **explicitly mentioned** the use of inclusive and accessible communications checklist produced by WP2 and the WP5 JACARDI terminology glossary for internal and external communication in the pilot implementation plan.	b) The pilot implementation plan had a **specific objective** related to inclusive and accessible communications for pilot internal and external communication.	c) The pilot implementation plan integrated **an action point** on inclusive and accessible communications for external and internal communication under each specific objective.	
**VII Roll-out of actions and monitoring**				
**7.1 Identify how equity and diversity principles will be monitored**	a) Plans for monitoring the principles of equity and diversity during roll-out of actions were identified based on **critical reflection** within the pilot team.	b) Plans for monitoring equity and diversity principles during roll-out were identified in **consultations** with diverse end users/end beneficiaries and other stakeholders.	c) Plans for monitoring equity and diversity principles during roll-out were identified in **co-design** with diverse end users/ end beneficiaries and other stakeholders and based on the pilot implementation plan.	
**7.2 Apply equity and diversity perspectives during roll-out of actions**	a) **Critical reflection** was applied within the core pilot team during roll-out of actions (e.g. how are different groups affected; are some groups unintentionally left behind; are relevant and diverse stakeholders meaningfully involved; are pilot communications inclusive and accessible for the target populations).	b) **Consultations** with diverse end users/ end beneficiaries and other stakeholders took place to support the roll-out of the project.	c) The roll-out was conducted in **co-design** with diverse end users/end beneficiaries and other stakeholders during the project's roll-out.	

Co-funded by the European Union

	Approaching	Meeting	Exceeding	Describe
**7.3 Continue strengthening capacity in equity and diversity within the pilot team**	a) The core pilot team members **understand and can explain** the 4Cs principle and the relevance of equity and diversity in JACARDI to other partners and this is demonstrated through integrating equity and diversity perspectives in the pilot implementation plan.	b) The core pilot team members understand how to include equity and diversity perspectives in their pilot implementation but **need support** (e.g. consultations, workshops) of equity and diversity subject matter experts on how to apply them in practice.	c) The core pilot team members feel empowered to apply sufficiently concrete equity and diversity aspects in their implementation plans in an **autonomous** manner, and **actively seek opportunities to strengthen their capacity** in equity and diversity beyond JACARDI structures, for example through external seminars, trainings and independent readings.	
**7.4 Consider core pilot team composition**	a) The core pilot team consists of professionals from **different disciplines and social groups**, representing at least two key characteristics of the community the pilot aims to serve.	b) The core pilot team consists of professionals from different disciplines and social groups (e.g. age, gender, ethnicity), and is **moderately representative** of the community the pilot aims to serve (i.e. 50% of key characteristics of the served community are represented within the team).	c) The core pilot team consists of professionals from diverse disciplines, social groups, and is **representative of the community** the pilot aims to serve (i.e. 75% or more of the key characteristics identified are represented within the team).	
**VIII Intermediate report No 1**				
**8.1 Engage diverse stakeholders in evaluation of intermediate results**	a) **Critical reflection** was applied within the core pilot team to consider whether there is a need for changes at action level or specific objectives levels that would further strengthen equity and diversity perspectives in the pilot, and these were **described and assessed** in the intermediate report. Relevant **stakeholders** were **informed** of key aspects of the intermediate report.	b) **Consultations** were conducted with diverse end users/ end beneficiaries and other stakeholders to consider whether there is a need for changes at general objectives or specific objectives level that would further strengthen equity and diversity perspectives in the pilot, and these are **described and assessed** in the intermediate report.	c) Diverse end users/ end beneficiaries and other stakeholders were meaningfully engaged through **co-design** to consider whether there is a need for changes at action level or specific objectives level that would further strengthen equity and diversity perspectives in the pilot, and these were **described and assessed** in the intermediate report.	

Co-funded by the European Union

	Approaching	Meeting	Exceeding	Describe
**8.2 Integrate equity and diversity perspectives in intermediate reporting**	a) Equity and diversity perspectives were reported under the **general objective**.	b) A **specific objective** related to equity and diversity (other than inclusive and accessible communications) was reported.	c) An **action point** related to equity and diversity (other than inclusive and accessible communications) was reported in relation to each specific objective.	
**8.3 Integrate equity and diversity perspectives in intermediate reporting on pilot communication**	a) The report **explicitly mentioned** the use of inclusive and accessible communications checklist produced by WP2 and the WP5 JACARDI terminology glossary for internal and external communication.	b) A **specific objective** related to inclusive and accessible communications for pilot internal and external communication was reported.	c) An **action point** on inclusive and accessible communications for external and internal communication was reported in relation to each specific objective.	
**IX Pilot implementation plan No 2**				
**9.1 Engage diverse stakeholders in development of pilot implementation plan**	a) Implementation plan No 2 compiled by the pilot team and stakeholder board was **informed** on relevant revisions of the pilot implementation plan.	b) Stakeholder board was **consulted** when developing the pilot implementation plan No 2.	c) Stakeholder board was meaningfully engaged through **co-design** when developing the pilot implementation plan No 2.	
**9.2 Integrate equity and diversity perspectives in the pilot implementation plan**	a) Equity and diversity perspectives were **integrated in the general objective** of the pilot implementation plan No 2.	b) The pilot implementation plan had at least one **specific objective** related to equity and diversity of the pilot implementation plan No 2.	c) The pilot implementation plan had **an action point** on equity and diversity under each specific objective of the pilot implementation plan No 2.	

Co-funded by the European Union

	Approaching	Meeting	Exceeding	Describe
**9.3 Integrate equity and diversity perspectives in the pilot communication**	a) The pilot implementation plan **explicitly mentioned** the inclusive and accessible communications checklist produced by WP2 and the WP5 JACARDI terminology glossary for internal and external communication in the pilot implementation plan.	b) The pilot implementation plan had a **specific objective** related to inclusive and accessible communications for pilot internal and external communication.	c) The pilot implementation plan integrated **an action point** on inclusive and accessible communications for external and internal communication under each specific objective.	
**X Roll out of actions and monitoring**				
**10.1 Monitor (and if needed revise) equity and diversity principles**	a) Equity and diversity perspectives monitored were identified based on **critical reflection** within the pilot team.	b) Equity and diversity perspectives monitored were identified in **consultations** with diverse end users/end beneficiaries and other stakeholders.	c) Equity and diversity perspectives monitored were identified in **co-design** with diverse end users/ end beneficiaries and other stakeholders.	
**10.2 Apply equity and diversity perspectives during roll-out of actions**	a) **Critical reflection** was applied within the core pilot team during roll-out of actions (e.g. how are different groups affected; are some groups unintentionally left behind; are relevant and diverse stakeholders meaningfully involved; are pilot communications inclusive and accessible for the target populations).	b) **Consultations** with diverse end users/ end beneficiaries and other stakeholder took place to support the roll-out of the project.	c) The roll-out was done in **co-design** with diverse end users/end beneficiaries and other stakeholders during the project's roll-out.	

	Approaching	Meeting	Exceeding	Describe
**10.3 Continue strengthening capacity in equity and diversity within the pilot team**	A) The core pilot team members **understand and can explain** the 4Cs principle and the relevance of equity and diversity in JACARDI to other partners and this is demonstrated through integrating equity and diversity perspectives in the pilot implementation plan.	b) The core pilot team members understand how to include equity and diversity perspectives in their pilot implementation but **need support** (e.g. consultations, workshops) of equity and diversity subject matter experts on how to apply them in practice.	c) The core pilot team members feel empowered to apply sufficiently concrete equity and diversity aspects in their implementation plans in an **autonomous** manner, and **actively seek opportunities to strengthen their capacity** in equity and diversity beyond JACARDI structures, for example through external seminars, trainings and independent readings.	
**10.4 Consider core pilot team composition**	a) The core pilot team consists of professionals from **different disciplines and social groups**, representing at least two key characteristics of the community the pilot aims to serve.	b) The core pilot team consists of professionals from different disciplines and social groups (e.g. age, gender, ethnicity), and is **moderately representative** of the community the pilot aims to serve (i.e. 50% of key characteristics of the served community are represented within the team).	c) The core pilot team consists of professionals from diverse disciplines, social groups, and is **representative of the community** the pilot aims to serve (i.e. 75% or more of the key characteristics identified are represented within the team).	
**XI Intermediate report No 2**				
**11.1 Engage diverse stakeholders in evaluation of intermediate results**	a) **Critical reflection** was applied within the core pilot team to consider whether there is a need for changes at action level or specific objectives levels that would further strengthen equity and diversity perspectives in the pilot, and these were **described and assessed** in the intermediate report. Relevant **stakeholders** were **informed** of key aspects of the intermediate report.	b) **Consultations** were conducted with diverse end users/ end beneficiaries and other stakeholders to consider whether there is a need for changes at general objectives or specific objectives level that would further strengthen equity and diversity perspectives in the pilot, and these are **described and assessed** in the intermediate report.	c) Diverse end users/ end beneficiaries and other stakeholders were meaningfully engaged through **co-design** to consider whether there is a need for changes at action level or specific objectives level that would further strengthen equity and diversity perspectives in the pilot, and these were **described and assessed** in the intermediate report.	

Co-funded by the European Union

	Approaching	Meeting	Exceeding	Describe
**11.2 Integrate equity and diversity perspectives in intermediate reporting**	a) Equity and diversity perspectives were reported under the **general objective**.	b) A **specific objective** related to equity and diversity (other than inclusive and accessible communications) was reported.	c) An **action point** related to equity and diversity (other than inclusive and accessible communications) was reported in relation to each specific objective.	
**11.3 Integrate equity and diversity perspectives in intermediate reporting on pilot communication**	a) The report **explicitly mentioned** the use of inclusive and accessible communications checklist produced by WP2 and the WP5 JACARDI terminology glossary for internal and external communication.	b) A **specific objective** related to inclusive and accessible communications for pilot internal and external communication was reported.	c) An **action point** on inclusive and accessible communications for external and internal communication was reported in relation to each specific objective.	
**XII Final implementation report**				
**12.1 Return results to the community and engage diverse stakeholders in evaluation of results**	a) Evaluation of results was conducted based on **critical reflection** within the core pilot team and relevant **stakeholders,** including end users and end beneficiaries were **informed** of results.	b) **Consultations** were conducted with diverse end users/ end beneficiaries when interpreting and evaluating results.	c) Results were interpreted and evaluated in **co-design** with diverse end users/ end beneficiaries.	
**12.2 Integrate equity and diversity perspectives in reporting**	a) Equity and diversity perspectives were reported under the **general objective**.	b) A **specific objective** related to equity and diversity (other than inclusive and accessible communications) was reported.	c) An **action point** related to equity and diversity (other than inclusive and accessible communications) was reported in relation to each specific objective.	

Co-funded by the European Union

	Approaching	Meeting	Exceeding	Describe
**12.3 Integrate equity and diversity perspectives in reporting on pilot communication**	a) The report **explicitly mentioned** the use of inclusive and accessible communications checklist produced by WP2 and the WP5 JACARDI terminology glossary for internal and external communication.	b) A **specific objective** related to inclusive and accessible communications for pilot internal and external communication was reported.	c) An **action point** on inclusive and accessible communications for external and internal communication was reported in relation to each specific objective.	
**XIII Focus on the key stakeholders' engagement in building sustainability**				
**13.1 Revisit (and if needed revise) the key stakeholder analysis**	a) **Critical reflection** applied within the core pilot team to review and revise the key stakeholders and their level of involvement, with consideration of diversity representation (e.g. experts and professionals, policy makers representing different disciplines and social groups, and persons with lived experiences).	b) **Consultations** conducted with diverse individuals, groups, or entities considered to be potentially directly or indirectly affected by the pilot to comprehensively review and revise relevant stakeholders.	c) Review and revision of key stakeholders carried out in **co-design** with diverse individuals, groups, or entities considered directly or indirectly affected by the pilot.	
**13.2 Apply equity and diversity perspectives in dissemination of results and sustainability actions**	a) **Critical reflection** was applied within the core pilot team to identify priorities for future implementation, plans for dissemination of pilot results and sustainability actions.	b) **Consultations** were conducted with diverse end users/ end beneficiaries and other stakeholders to identify priorities for future implementation, plans for dissemination of pilot results and sustainability actions.	c) Priorities for future implementation, plans for dissemination of pilot results and sustainability actions were identified in **co-design** with diverse end users/ end beneficiaries and other stakeholders.	

Co-funded by
the European Union

	Approaching	Meeting	Exceeding	Describe
**13.3 Apply inclusivity and accessibility guidelines in material presented to the stakeholder board**	a) **Critical reflection** was applied within the core pilot team in development of materials presented to the stakeholder board, following the WP2 guidelines for inclusive and accessible communications.	b) **Consultations** with diverse end users/ end beneficiaries and other stakeholders were conducted during development of materials presented to the stakeholder board, following the WP2 guidelines for inclusive and accessible communications.	c) Materials presented to the stakeholder board were developed in **co-design** with diverse end users/ end beneficiaries and other stakeholders, following the WP2 guidelines for inclusive and accessible communications.	
**XIV Sustainability action plan**				
**14.1 Apply equity and diversity perspectives in development of the sustainability action plan**	a) **Critical reflection** was applied within the core pilot team in developing the sustainability action plan, including consideration of impact and inclusivity of planned actions for diverse population groups.	b) The sustainability action plan was developed in **consultations** with diverse end users/ end beneficiaries and other stakeholders, including consideration of impact and inclusivity of planned actions for diverse population groups.	c) The sustainability action plan was developed in **co-design** with diverse end users/ end beneficiaries and other stakeholders, including consideration of impact and inclusivity of planned actions for diverse population groups.	
**14.2 Integrate equity and diversity perspectives in the sustainability action plan**	a) Equity and diversity perspectives were **integrated in the general objective of** the sustainability action plan.	b) The sustainability action plan had at least one **specific objective** related to equity and diversity.	c) The sustainability action plan plan had **an action point** on equity and diversity under each specific objective.	
**14.3 Integrate inclusive and accessible communications in the sustainability action plan**	a) The sustainability action plan **explicitly mentioned** the use of inclusive and accessible communications checklist produced by WP2 and the WP5 JACARDI terminology glossary for internal and external communication in the pilot implementation plan.	b) The sustainability action plan had a **specific objective** related to inclusive and accessible communications for pilot internal and external communication.	c) The sustainability action plan integrated **an action point** on inclusive and accessible communications for external and internal communication under each specific objective.	

	Approaching	Meeting	Exceeding	Describe
**XV Celebrate the success**				
**15.1 Engage diverse stakeholders in planning final dissemination events**	a) Diverse end users/ end beneficiaries and other stakeholders were **invited** into final dissemination events.	b) Diverse end users/ end beneficiaries and other stakeholders were **consulted** when planning final dissemination events.	c) Final dissemination events were **co-designed** with diverse end users/ end beneficiaries and other stakeholders, and end users/ end beneficiaries were included in pilot publications (if any).	
**15.2 Consider representation among the speakers in the final dissemination events**	a) Speakers at the final dissemination events represented **different disciplines and social groups**, representing at least two key characteristics of the community the pilot aims to serve.	b) Speakers at the final dissemination events were **moderately representative** of the community the pilot aims to serve (i.e. 50% of key characteristics of the served community are represented within the team).	c) Speakers at the final dissemination events were **representative of the community** the pilot aims to serve (i.e. 75% or more of the key characteristics identified are represented within the team).	
**15.3 Apply the principles of inclusive and accessible communications in final dissemination events**	a) **Critical reflection** was applied within the core pilot team when planning and conducting final dissemination events, following the WP2 guidelines for inclusive and accessible communications.	b) Diverse stakeholders were **consulted** when planning and conducting final dissemination events, following WP2 guidelines for inclusive and accessible communications.	c) Planning and conducting of final dissemination events were performed in **co-design** with diverse key stakeholders, following WP2 guidelines for inclusive and accessible communications.	

JACARDI

This project has received funding from the EU4Health Programme 2021-2027 under Grant Agreement 101126953. Views and opinions expressed are however those of the author(s) only and do not necessarily reflect those of the European Union or the European Health and Digital Executive Agency (HaDEA). Neither the European Union nor the granting authority can be held responsible for them.

Co-funded by the European Union
