# Supplementary material for: Embedding equity and diversity principles in a complex multinational setting: methods, tools, capacity development and experiences from the first year of the Joint Action on Cardiovascular Diseases and Diabetes (JACARDI)
Source: BMJ Glob Health. 2025 Nov 16;10(11):e019829. doi: 10.1136/bmjgh-2025-019829 (PMC12625843; doi:10.1136/bmjgh-2025-019829)
Supplement: Supplementary file 2 [file bmjgh-10-11-s002.docx]

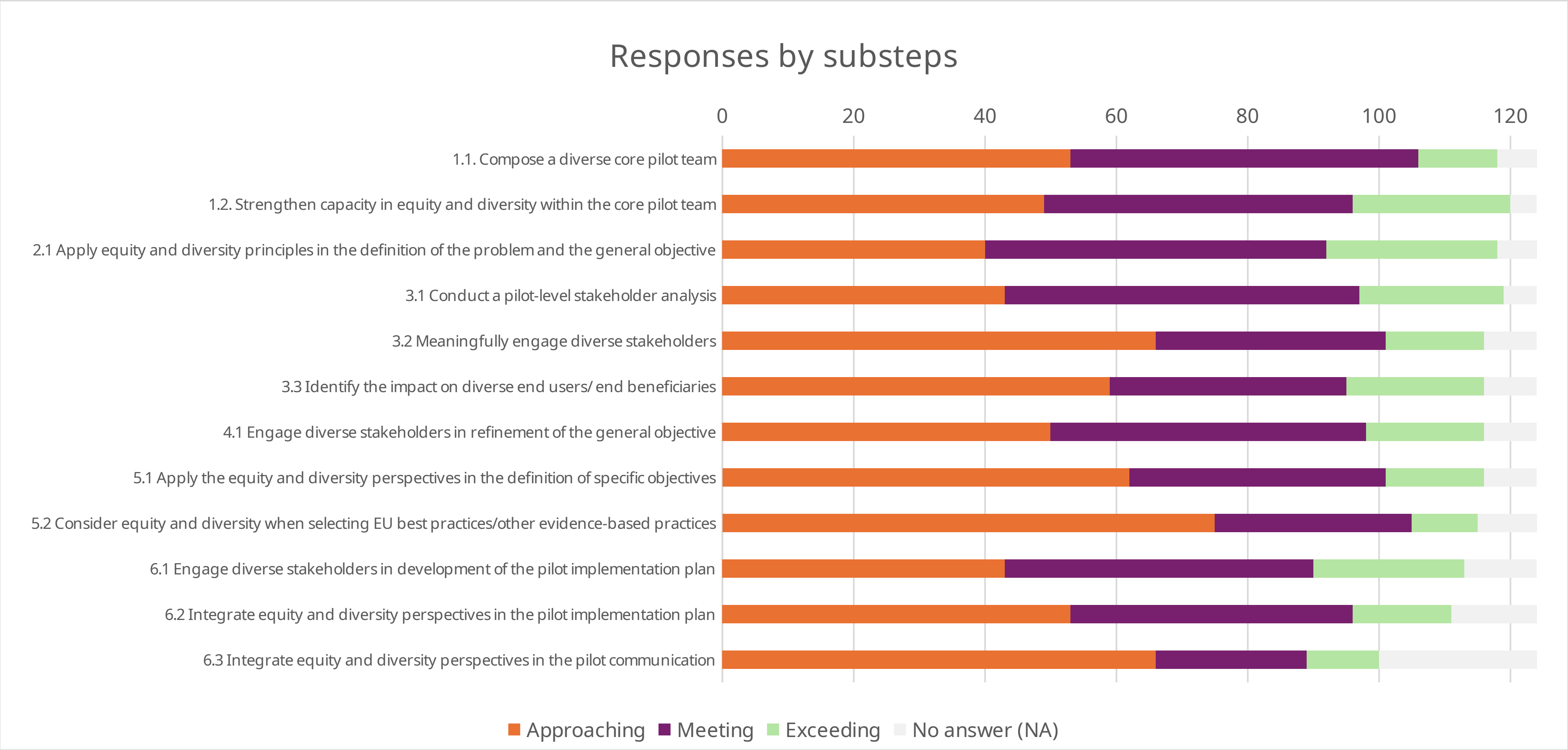

Responses by substeps
0
20
40
60
80
100
120
1.1. Compose a diverse core pilot team
1.2. Strengthen capacity in equity and diversity within the core pilot team
2.1 Apply equity and diversity principles in the definition of the problem and the general objective
3.1 Conduct a pilot-level stakeholder analysis
3.2 Meaningfully engage diverse stakeholders
3.3 Identify the impact on diverse end users/ end beneficiaries
4.1 Engage diverse stakeholders in refinement of the general objective
5.1 Apply the equity and diversity perspectives in the definition of specific objectives
5.2 Consider equity and diversity when selecting EU best practices/other evidence-based practices
6.1 Engage diverse stakeholders in development of the pilot implementation plan
6.2 Integrate equity and diversity perspectives in the pilot implementation plan
6.3 Integrate equity and diversity perspectives in the pilot communication
Approaching
Meeting
Exceeding
No answer (NA)
